# Pharmaceutical services: opportunities and perspectives for generating scientific evidence

**DOI:** 10.1590/S2237-96222025v34e20252003.en

**Published:** 2025-11-10

**Authors:** Maria Auxiliadora Parreiras Martins, Jorge Otávio Maia Barreto, Everton Nunes da Silva, Taís Freire Galvão

**Affiliations:** 1 Universidade Federal de Minas Gerais, Faculdade de Farmácia, Belo Horizonte, MG, Brazil; 2 Fundação Oswaldo Cruz, Gerência Regional de Brasília, Brasília, DF, Brazil; 3 Universidade de Brasília, Faculdade de Ciências e Tecnologias em Saúde, Brasília, DF, Brazil; 4 Universidade Estadual de Campinas, Faculdade de Ciências Farmacêuticas, Campinas, SP, Brazil

Pharmaceutical services play a fundamental role in the care of health service users at all levels of complexity. Their incorporation as a public policy in the Brazilian Unified Health System (*Sistema Único de Saúde*, SUS) paved the way for advances in the organizational arrangements of the Brazilian healthcare system and strengthened linkage with the population through improved access to and availability of medications ^1^. However, the scope and objectives of pharmaceutical services are comprehensive and transcend the focal context of medication supply. Pharmaceutical service practices are also relevant for ensuring the rational use of these therapeutic resources, including their appropriate administration according to the clinical and individual needs of each patient, aiming to achieve effectiveness, safety and convenience of the dosage regimen ^2^ ^,^ ^3^. 

In pharmaceutical service practice, there are numerous challenges to achieving pharmacological goals with acceptable safety and cost. It is important to consider access indicators that involve diagnosis, therapeutic options, supply, cost, regional aspects, among others. Individual factors can also influence the medication use process, including the following: sex, age, lifestyle, socioeconomic status, level of knowledge about the disease and treatment, level of health literacy, beliefs, attitudes, motivation, family dynamics, patient engagement in self-care and adherence to drug therapy. Drug therapy monitoring is therefore necessary, both in outpatient and inpatient settings, to identify and manage medication-related problems. Thus, clinical pharmaceutical services can make significant contributions to reducing morbidity and mortality related to such problems and improving the medication user experience ^4^.

Medication-related problems include adverse reactions, drug interactions, medication errors, patient non-adherence and self-medication. Given the rapid aging of the population, this scenario becomes even more complex due to the high prevalence of chronic diseases and consequent polypharmacy ^4^. In addition to the risks for certain subgroups (e.g. the very young and the very old, pregnant women, people with kidney or liver dysfunction), some therapeutic classes or specific medications present higher risk of serious or fatal clinical complications when used inappropriately or involved in medication errors, and are known as potentially dangerous medications (e.g. antithrombotics, chemotherapy drugs and insulin) ^5^. Prevention and management of adverse drug events requires coordinated actions and a multidisciplinary approach, involving health service managers, healthcare professionals, patients and caregivers. This approach demands a high level of commitment from the healthcare system and the strengthening of practices guided by the best available evidence, to continuously improve these processes and establish a culture of safety with a broad and systemic vision, aligned with the guidelines of the National Patient Safety Program_^(6)^_ .

Given the challenges outlined, pharmaceutical service research in Brazil takes on great importance, as it contributes to a better understanding of drug utilization processes in the country. Epidemiological methods are highly useful tools for characterizing utilization, effectiveness, risks, costs, and other dimensions when drugs are used on a large scale, that is, in the post-marketing phase. Study design with methodological rigor allows for the generation of scientific evidence from data collected in healthcare services, which can feed back into the system with reliable results to support decision-making and the development of strategies focused on promoting the rational use of medications. Such studies can also contribute to the understanding of regional particularities and challenges in the SUS. 

Research in this area has been increasingly important for the national and international scientific community. Topics related to pharmaceutical services are within the scope of Epidemiology and Health Services: Journal of the Brazilian National Health System (*Epidemiologia e Serviços de Saúde: revista do SUS* - RESS), which seeks to disseminate public health knowledge and relevant evidence to researchers, students, health service managers and health professionals. The number of publications in this field has also been growing recently in RESS, although there is room for expansion. In recent years, research results in the field of pharmaceutical services have been published, covering medication storage ^7^, dispensing ^8^, access ^9^ ^,^ ^10^, costs ^11^, medication use ^12^ ^,^ ^13^, patient safety ^14^ ^,^ ^15^, antimicrobial resistance ^16^, polypharmacy ^17^ ^,^ ^18^, and self-medication ^19^, in studies that address various realities of the SUS.

Among the study designs and themes that could be further explored, we highlight systematic reviews and analytical observational studies based on service data, including those using telehealth tools. Qualitative methods employed in pharmaceutical service research allow for a better understanding of behaviors and subjectivities related to the illness and treatment process from the perspective of health service users, caregivers and healthcare professionals. Infectious diseases and antimicrobial resistance are also topics of great interest due to their impact on public health. 

RESS is committed to increasing the dissemination of such evidence and encourages the academic and healthcare community to submit studies that explore the various dimensions of medication use. As such, it is essential to develop research with high standards of quality, ethics and transparency, fostering in-depth reflection on its impact and implications for the SUS. Studies on medications in populations need to follow these premises to strengthen pharmaceutical services in Brazil and consolidate their social impact.
