# Supplementary material for: Pharmaceutical services: opportunities and perspectives for generating scientific evidence
Source: Epidemiol Serv Saude. 2025 Nov 10;34:e20252003. doi: 10.1590/S2237-96222025v34e20252003.en (PMC12599862; doi:10.1590/S2237-96222025v34e20252003.en)
Supplement: Translation

# Translation: Assistência farmacêutica: oportunidades e perspectivas para geração de evidências científicas

**DOI:** 10.1590/S2237-96222025v34e20252003.pt

**Authors:** Maria Auxiliadora Parreiras Martins, Jorge Otávio Maia Barreto, Everton Nunes da Silva, Taís Freire Galvão

**Affiliations:** 1 Universidade Federal de Minas Gerais, Faculdade de Farmácia, Belo Horizonte, MG, Brasil; 2 Fundação Oswaldo Cruz, Gerência Regional de Brasília, Brasília, DF, Brasil; 3 Universidade de Brasília, Faculdade de Ciências e Tecnologias em Saúde, Brasília, DF, Brasil; 4 Universidade Estadual de Campinas, Faculdade de Ciências Farmacêuticas, Campinas, SP, Brasil

A assistência farmacêutica desempenha papel fundamental no cuidado aos usuários dos serviços de saúde, nos diferentes níveis de complexidade. Sua incorporação como política pública no Sistema Único de Saúde (SUS) pavimentou avanços nos arranjos organizativos do sistema de saúde brasileiro e fortaleceu vínculos com a população, por meio do acesso a medicamentos e a disponibilidade destes ^1^. Todavia, o escopo e os objetivos da assistência farmacêutica são abrangentes e transcendem o contexto focal do abastecimento de medicamentos. Suas práticas são, também, relevantes para assegurar o uso racional desses recursos terapêuticos, compreendendo a sua administração adequada, de acordo com as necessidades clínicas e individuais de cada paciente, visando à efetividade, segurança e comodidade do regime posológico ^2^ ^,^ ^3^. 

Na prática assistencial, há inúmeros desafios para alcance das metas farmacológicas, com segurança e custo aceitáveis. É importante considerar indicadores de acesso que envolvam diagnóstico, opções terapêuticas, abastecimento, custo, aspectos regionais, entre outros. Fatores individuais também podem interferir no processo de utilização de medicamentos, podendo-se destacar os seguintes: sexo, idade, estilo de vida, nível socioeconômico, grau de conhecimento sobre a doença e o tratamento, nível de letramento em saúde, crenças, atitudes, motivação, dinâmica familiar, engajamento do paciente em seu autocuidado e adesão à farmacoterapia. O acompanhamento farmacoterapêutico faz-se então necessário, tanto em nível ambulatorial quanto hospitalar, para identificar e manejar os problemas relacionados aos medicamentos. Dessa forma, os serviços clínicos farmacêuticos podem trazer grandes contribuições para reduzir a morbimortalidade relacionada a tais problemas e melhorar a experiência do usuário do medicamento ^4^.

Os problemas relacionados aos medicamentos abrangem reações adversas, interações medicamentosas, erros de medicação, não adesão do paciente e automedicação. Diante do envelhecimento populacional acelerado, esse cenário se torna ainda mais complexo, devido à alta prevalência de doenças crônicas e consequente polifarmácia ^4^. Além dos riscos para certos subgrupos (por exemplo, extremos etários, gestantes, pessoas com disfunção renal ou hepática), algumas classes terapêuticas ou medicamentos específicos apresentam maior risco de complicações clínicas graves ou fatais quando utilizados de forma inadequada ou envolvidos em erros de medicação, sendo conhecidos como medicamentos potencialmente perigosos (a exemplo de antitrombóticos, quimioterápicos e insulina) ^5^. A prevenção e manejo de eventos adversos relacionados aos medicamentos requer ações articuladas e abordagem multiprofissional, com envolvimento de gestores, profissionais de saúde, pacientes e cuidadores. Essa atuação exige do sistema de saúde elevado grau de comprometimento e fortalecimento das práticas guiadas pelas melhores evidências disponíveis, para aprimorar continuamente estes processos e estabelecer uma cultura de segurança com visão ampliada e sistêmica, alinhada com as diretrizes do Programa Nacional de Segurança do Paciente ^6^.

Diante dos desafios expostos, a pesquisa no âmbito da assistência farmacêutica no Brasil assume grande importância, na medida em que contribui com a melhor compreensão dos processos de utilização de medicamentos no país. Os métodos epidemiológicos são ferramentas de grande utilidade para caracterizar a utilização, a efetividade, os riscos, os custos, entre outras dimensões, quando os medicamentos são utilizados em larga escala, ou seja, na fase pós-comercialização. O delineamento de estudos com rigor metodológico permite a geração de evidências científicas a partir de dados coletados nos serviços de saúde, os quais podem retroalimentar o sistema com resultados confiáveis, para subsidiar a tomada de decisão e a elaboração de estratégias focadas na promoção do uso racional de medicamentos. Esses estudos podem, ainda, contribuir para o entendimento das particularidades regionais e dos desafios no SUS. 

Tem sido observado um papel cada vez mais importante das pesquisas desenvolvidas nesta área pela comunidade científica nacional e internacional. As temáticas relacionadas à assistência farmacêutica vão ao encontro do escopo da *Epidemiologia e Serviços de Saúde: revista do SUS* (RESS), que busca divulgar o conhecimento e as evidências relevantes em saúde coletiva para pesquisadores, estudantes, gestores e profissionais de saúde. O número de publicações nesse campo tem crescido ultimamente, também, na RESS, ainda que haja espaço para expansão. Nos últimos anos, foram publicados resultados de pesquisas no âmbito da assistência farmacêutica, compreendendo armazenamento ^7^, dispensação de medicamentos ^8^, acesso ^9^ ^,^ ^10^, custos ^11^, utilização de medicamentos ^12^ ^,^ ^13^, segurança do paciente ^14^ ^,^ ^15^, resistência microbiana ^16^, polifarmácia ^17^ ^,^ ^18^ e automedicação ^19^, em pesquisas que trazem diferentes realidades do SUS.

Entre delineamentos e temáticas que podem ser mais explorados, destacamos revisões sistemáticas e estudos observacionais analíticos oriundos de dados de serviços, inclusive com ferramentas de telessaúde. Métodos qualitativos empregados em pesquisas na área de assistência farmacêutica permitem maior compreensão dos comportamentos e das subjetividades frente ao processo de adoecimento e tratamento na perspectiva dos usuários, cuidadores e profissionais de saúde. Doenças infecciosas e resistência aos antimicrobianos também constituem temáticas de grande interesse, devido ao seu impacto na saúde pública. 

A RESS se coloca à disposição para aumentar a disseminação de tais evidências, e incentiva a comunidade acadêmica e de serviços de saúde a submeterem estudos que explorem as várias dimensões da utilização de medicamentos. Nesse sentido, é essencial o desenvolvimento de pesquisas com elevado padrão de qualidade, ética e transparência, fomentando reflexão aprofundada sobre seu impacto e suas implicações para o SUS. Estudos sobre medicamentos em populações devem seguir tais premissas, para o fortalecimento da assistência farmacêutica no Brasil e a consolidação do seu impacto social.
